# Disease Burden of Gastrointestinal Tumors in China From 1990 to 2021, an Analysis for the Global Burden of Disease Study 2021

**DOI:** 10.1111/jebm.70072

**Published:** 2025-09-20

**Authors:** Lanwei Guo, Jiani Yuan, Lin Cai, Chenxin Zhu, Yan Zheng, Haiyan Yang, Yanyan Liu

**Affiliations:** ^1^ Department of Clinical Research Management The Affiliated Cancer Hospital of Zhengzhou University & Henan Cancer Hospital Zhengzhou China; ^2^ Department of Epidemiology, School of Public Health Zhengzhou University Zhengzhou China

**Keywords:** burden, gastrointestinal tumors, trends

## Abstract

**Objective:**

China faces a significant burden of gastrointestinal tumors driven by socioeconomic, environmental, and lifestyle factors. Using GBD2021 data, this study analyses epidemiological trends and disease burden for six major gastrointestinal tumor cancers (esophagus, gastric, colorectum, liver, pancreas, gallbladder and biliary tract) in China (1990–2021).

**Methods:**

The GBD 2021 was used to extract the incidence, mortality, and disability‐adjusted life years (DALYs) data of gastrointestinal tumors in China. Age‐standardized rates (ASRs) and 95% uncertainty intervals (UIs) were calculated. Temporal trends were assessed by joinpoint regression analysis, and average annual percent change (AAPC) and annual percentage change (APC) were calculated and analyzed stratified by gender and age group.

**Results:**

In 2021, China recorded 1.96 million new gastrointestinal cancer cases, with 1.35 million deaths and 33.07 million DALYs. Gastric cancer led in mortality, and colorectal cancer demonstrated the most rapid incidence growth (AAPC = 1.68). Significant reductions were observed in gastric cancer age‐standardized mortality rates (ASMR) (AAPC = –2.44) and esophageal cancer age‐standardized disability‐adjusted life year rates (ASDR) (AAPC = –2.31). Gender disparities were particularly pronounced in esophageal cancer, with the male‐to‐female mortality ratio (M/F) escalating from 2.50 (1990) to 4.12 (2021). The age group with the highest mortality burden was 70–74, while the age group with the most significant loss of DALYs was 65–69.

**Conclusion:**

China has significantly reduced gastrointestinal cancer burden, but gender and age disparities persist, necessitating targeted interventions. Future efforts should focus on tertiary prevention for high‐risk groups, especially males and the elderly, while enhancing molecular subtyping and regional data stratification for precision cancer control.

## 1 Introduction

Cancer is the second leading cause of death worldwide, and it is predicted that the global cancer burden will increase over the next 20 years [1]. Gastrointestinal tumors are malignant tumors in the digestive tract and digestive system (including the esophagus, gastric, colorectum, liver, pancreas, and gallbladder and biliary tract). It has been estimated that gastrointestinal tumors constitute about 25% of global malignant tumor incidence and contribute to nearly one‐third of all cancer‐associated mortality [2]. Consequently, the burden of disease resulting from these tumors has become a significant challenge for global public health. The epidemiology of gastrointestinal tumors displays significant geographic heterogeneity. East Asia carries the highest burden of gastric, liver, and esophageal malignancies, while Western Europe demonstrates elevated pancreatic cancer rates, and Southeast Asia shows a progressive rise in colorectal cancer incidence [3].

Over the past few decades, the epidemiology of gastrointestinal tumors in China, the world's largest developing country, has undergone a series of dynamic changes due to economic globalization, environmental changes, and shifting dietary patterns. The objective of this study was to utilize the most recent iteration of the Global Burden of Disease (GBD) 2021 to characterize the burden of gastrointestinal tumors in China from 1990 to 2021, with particular focus on disparities across sex and age groups as well as temporal trends.

## 2 Materials and Methods

### 2.1 Data Source

According to the International Classification of Diseases 10th edition (ICD‐10), the cancer codes for the six types of gastrointestinal tumors are delineated as follows: esophageal (C15), gastric (C16), liver (C22), gallbladder and biliary tract (C23–24), pancreatic (C25), and colorectal (C18–21) cancers.

The Chinese gastrointestinal tumors data analyzed in this study were obtained from GBD 2021, which provides up‐to‐date estimates of epidemiological data for 371 disease and injury burdens across 21 GBD regions and 204 countries from 1990 to 2021. Detailed information on the relevant data, methods, and statistical models it provides can be found in previous reports [4]. This study used GHDx (http://ghdx. healthdata.org/gbd‐results‐tool) to collect data on incidence, mortality, and disability‐adjusted life‐years (DALYs) due to gastrointestinal tumors in China. Incidence estimates provided by GBD were generated using DisMod‐MR 2.1, which is a Bayesian disease modelling meta‐regression tool [5]. Mortality estimates were generated using another modelling tool developed by GBD (CODEm), which generates cause‐specific mortality estimates by evaluating the out‐of‐sample predictive validity of different combinations of statistical models and covariates and integrating the optimal results. DALYs is the sum of years of life lost (YLL), years lived with disability (YLD), which quantifies the health loss due to a specific disease [5]. YLLs are calculated by multiplying the number of cause‐sex‐location‐year‐specific deaths by the standardized life expectancy at the age at which the death occurred, and YLD is calculated by combining the cause‐age‐sex‐location‐specific prevalence of sequelae for each disease and injury times their respective disability weights [4].

### 2.2 Statistical Analysis

Absolute numbers and age‐standardized rates (ASRs) of gastrointestinal tumors incidence, death, and DALYs in China were extracted annually from 1990 to 2021, and estimates of the incidence of new cases and deaths were reported with 95% uncertainty intervals (UIs) computed as the 2.5 and 97.5 ranked values of the 1000th posterior distributions. The ASRs uses calculations with the standardized population of the GBD world population to adjust the age structure [6, 7]. Data were stratified by sex and 20 age groups (5‐year intervals from 0 to 95 years). Trends in the gastrointestinal tumors burden in China from 1990 to 2021 were assessed using joinpoint regression analysis, version 5.4.0 (National Cancer Institute, USA), a methodology that supports segmented regression modeling based on the characteristics of the temporal distribution of disease, and that facilitates trend fitting and optimization of each interval by dividing the study period into different intervals using multiple connected points [8]. The model was constructed using a grid search method (GSM), with the maximum number of joinpoints set to 5, and optimized through the default permutation test method. The joinpoint regression analysis was used to calculate the average annual percent change (AAPC), annualized percentage change (APC), and corresponding 95% confidence intervals (95% CI) for this study. APC was used to detect a linear trend for a specific stage and the calculation formula is $APC=\left(\frac{Y_{\left(x+1\right)}-Y_{x}}{Y_{x}}\right)\times 100\%\;=\left(e^{\beta_{1}}-1\right)\times 100\%$, where *Y* represents the age‐standardized rate, *x* represents the calendar year, *e* represents the natural logarithm, and *β*_1_ is the regression coefficient, while AAPC was used to estimate the mean APC for the entire period. When both the annualized rate of change and the lower bound of the 95% CI are positive, this is considered to be a growing trend. Conversely, when both the annualized rate of change and the upper limit were negative, this was considered a downward trend. In addition, R (version 4.3.0) was used for data analysis and visualization, with statistical significance set at P<0.05.

## 3 Results

### 3.1 Overall Disease Burden

According to GBD 2021, there were 1,957,947 new cases of gastrointestinal tumors in China in 2021, of which 1,358,958 (69.4%) were males and 598,989 (30.6%) were females (Table 1). The estimated number of deaths attributable to gastrointestinal tumors was 1,346,089, including 936,080 (69.5%) male cases and 410,009 (30.5%) female cases (Table 2). Gastrointestinal tumors resulted in a loss of approximately 33.07 million disability‐adjusted life years (DALYs) in the Chinese population, of which 23.86 million (72.1%) were borne by males and 9.21 million (27.9%) were borne by females (Table 3). In 2021, the ranking of gastrointestinal tumors by highest incidence rates was: colorectal cancer, gastric cancer, esophageal cancer, liver cancer, pancreatic cancer, and gallbladder and biliary tract cancers (Table 1). When ranked by mortality and DALY, the order became: gastric cancer, esophageal cancer, colorectal cancer, liver cancer, pancreatic cancer, and gallbladder and biliary tract cancers (Tables 2 and 3).

**TABLE 1:** The incident number and ASIR in 1990 and 2021 and its temporal trends.

Cancer type	1990 Incidence number (95% UI)	2021 Incidence number (95% UI)	1990 ASIR per 100,000 (95% UI)	2021 ASIR per 100,000 (95% UI)	AAPC (95% CI)
**Gastric cancer**					
Both	407,471 (337,565, 477,569)	611,799 (471,966, 765,562)	48.03 (40.21, 56.69)	29.05 (22.42, 36.20)	−1.61 (–1.74, –1.49)
Male	278,596 (208,188, 346,574)	446,434 (325,932, 589,284)	67.64 (51.71, 83.67)	44.48 (32.18, 58.38)	−1.34 (–1.52, –1.17)
Female	128,875 (105,159, 157,699)	165,365 (127,716, 208,140)	30.22 (24.80, 36.89)	15.23 (11.77, 19.16)	−2.17 (–2.33, –2.02)
M/F	2.16	2.70	2.24	2.92	
**Liver cancer**					
Both	96,434 (80,971, 113,769)	196,637 (158,273, 243,558)	10.58 (8.94, 12.43)	9.52 (7.72, 11.78)	−0.31 (–0.39, –0.23)
Male	70,209 (56,752, 85,768)	143,788 (108,927, 193,831)	15.06 (12.20, 18.24)	14.34 (10.93, 19.18)	−0.10 (–0.17, –0.04)
Female	26,225 (20,941, 31,755)	52,848 (41,045, 67,026)	6.04 (4.82, 7.28)	4.89 (3.82, 6.18)	−0.64 (–0.74, –0.54)
M/F	2.68	2.72	2.49	2.93	
**Esophagus cancer**					
Both	207,495 (172,674, 241,459)	320,805 (256,102, 394,756)	24.80 (20.71, 28.73)	15.04 (12.04, 18.43)	−1.60 (–1.78, –1.42)
Male	147,558 (120,388, 177,779)	249,647 (191,966, 315,899)	36.07 (29.88, 42.92)	24.78 (19.19, 31.20)	−1.18 (–1.39, –0.98)
Female	59,937 (31,551, 75,277)	71,158 (41,895, 93,895)	14.45 (7.79, 18.00)	6.40 (3.76, 8.47)	−2.58 (–2.73 –2.43)
M/F	2.46	3.51	2.50	3.87	
**Pancreatic cancer**					
Both	37,818 (31,791, 44,068)	118,665 (94,623, 144,663)	4.54 (3.84, 5.29)	5.64 (4.52, 6.84)	0.72 (0.50, 0.94)
Male	22,555 (18,204, 27,474)	72,280 (54,334, 92,975)	5.55 (4.55, 6.64)	7.29 (5.55, 9.24)	0.89 (0.68, 1.10)
Female	15,262 (12,235, 18,866)	46,386 (34,923, 59,339)	3.64 (2.93, 4.48)	4.18 (3.15, 5.34)	0.48 (0.22, 0.74)
M/F	1.48	1.56	1.52	1.74	
**Colorectal cancer**					
Both	158,389 (135,419, 182,577)	658,321 (531,995, 798,063)	19.04 (16.46, 21.81)	31.44 (25.53, 37.97)	1.68 (1.55, 1.80)
Male	88,370 (69,951, 107,467)	419,012 (319,825, 541,668)	22.31 (17.92, 26.82)	42.24 (32.56, 54.26)	2.08 (1.95, 2.22)
Female	70,019 (55,493, 85,557)	239,309 (181,750, 305,839)	16.43 (13.18, 19.95)	21.87 (16.58, 27.92)	0.94 (0.72, 1.17)
M/F	1.26	1.75	1.36	1.93	
**Gallbladder and biliary tract cancers**					
Both	17,077 (13,003, 21,744)	51,720 (35,618, 66,848)	2.19 (1.68, 2.79)	2.49 (1.71, 3.21)	0.40 (0.22, 0.59)
Male	8138 (5378, 11,476)	27,797 (16,110, 38,966)	2.24(1.46, 3.23)	2.89 (1.69, 4.00)	0.81 (0.56, 1.07)
Female	8940 (6147, 12,548)	23,923 (14,698, 32,562)	2.19 (1.51, 3.05)	2.17 (1.33, 2.95)	−0.03 (–0.27, 0.22)
M/F	0.91	1.16	1.02	1.33	

Abbreviations: AAPC, average annual percentage change; ASIR, age‐standardized incidence rates; CI, confidence interval; UI, uncertainty interval.

**TABLE 2:** The death number and ASMR in 1990 and 2021 and its temporal trends.

Cancer type	1990 Death number (95% UI)	2021 Death number (95% UI)	1990 ASMR per 100,000 (95% UI)	2021 ASMR per 100,000 (95% UI)	AAPC (95% CI)
**Gastric cancer**					
Both	374,066 (310,921, 442,251)	445,013 (344,736, 555,834)	46.05 (38.88, 54.43)	21.51 (16.66, 26.61)	−2.44 (–2.62, –2.26)
Male	251,602 (188,204, 314,409)	314,779 (230,725, 418,722)	64.67 (49.60, 79.93)	32.61 (23.61, 42.80)	−2.21 (–2.45, –1.98)
Female	122,464 (100,701, 149,718)	130,234 (100,509, 163,561)	29.81 (24.69, 36.43)	12.02 (9.29, 15.10)	−2.90 (–3.05, –2.74)
M/F	2.05	2.42	2.17	2.71	
**Liver cancer**					
Both	94,937 (79,884, 111,527)	172,068 (139,621, 212,496)	10.75 (9.12, 12.61)	8.35 (6.80, 10.29)	−0.68 (–1.25, –0.10)
Male	68,304 (55,235, 83,128)	122,463 (93,115, 164,816)	15.19 (12.32, 18.36)	12.40 (9.46, 16.55)	−0.60 (–1.08, –0.12)
Female	26,633 (21,350, 32,258)	49,605 (38,617, 62,668)	6.33 (5.08, 7.64)	4.57 (3.57, 5.76)	−0.92 (–1.40, –0.44)
M/F	2.56	2.47	2.40	2.71	
**Esophagus cancer**					
Both	210,821 (176,081, 244,587)	296,443 (236,648, 362,831)	26.06 (21.77, 30.10)	14.13 (11.36, 17.18)	−1.51 (–1.71, –1.31)
Male	149,418 (122,412, 179,467)	232,754 (178,778, 294,009)	38.20 (31.85, 45.33)	23.84 (18.51, 29.79)	−3.10 (–3.37, –2.84)
Female	61,402 (32,748, 76,357)	63,689 (37,535, 83,584)	15.25 (8.35, 18.88)	5.79 (3.39, 7.58)	−1.98 (–2.19, –1.77)
M/F	2.43	3.65	2.50	4.12	
**Pancreatic cancer**					
Both	38,883 (32,790, 45,260)	119,602 (95,654, 145,218)	4.83 (4.10, 5.61)	5.72 (4.59, 6.91)	0.56 (0.31, 0.82)
Male	22,937 (18,492, 27,879)	72,159 (54,384, 92,255)	5.91 (4.88, 7.06)	7.37 (5.64, 9.30)	0.71 (0.49, 0.93)
Female	15,946 (12,876, 19,707)	47,443 (35,778, 60,492)	3.90 (3.15, 4.81)	4.29 (3.23, 5.46)	0.34 (0.06, 0.61)
M/F	1.44	1.52	1.52	1.72	
**Colorectal cancer**					
Both	119,304 (102,706, 137,153)	275,129 (223,379, 330,960)	15.49 (13.43, 17.70)	13.64 (11.09, 16.31)	−0.42 (–0.58, –0.25)
Male	66,235 (52,779, 80,322)	174,400 (133,842, 226,280)	18.56 (15.05, 22.07)	18.95 (14.65, 24.34)	0.01 (–0.23, 0.24)
Female	53,068 (42,217, 64,519)	100,729 (76,598, 128,091)	13.23 (10.70, 16.02)	9.34 (7.10, 11.88)	−1.11 (–1.31, –0.91)
M/F	1.25	1.73	1.40	2.03	
**Gallbladder and biliary tract cancers**					
Both	17,251 (13,213, 22,143)	37,834 (26,653, 49,262)	2.32 (1.78, 2.97)	1.85 (1.29, 2.40)	−0.73 (–0.94, –0.52)
Male	8081 (5345, 11,612)	19,525 (11,615, 27,421)	2.38 (1.55, 3.50)	2.11 (1.26, 2.92)	−0.42 (–0.78, –0.05)
Female	9170 (6355, 12,939)	18,309 (11,423, 24,764)	2.32 (1.60, 3.24)	1.67 (1.04, 2.25)	1.04 (–1.28, –0.80)
M/F	0.88	1.07	1.03	1.26	

Abbreviations: AAPC, average annual percentage change; ASMR, age‐standardized mortality rates; CI, confidence interval; UI, uncertainty interval.

**TABLE 3:** The DALY number and ASDR in 1990 and 2021 and its temporal trends.

Cancer type	1990 DALY number (95% UI)	2021 DALY number (95% UI)	1990 ASDR per 100,000 (95% UI)	2021 ASDR per 100,000 (95% UI)	AAPC (95% CI)
**Gastric cancer**					
Both	10,773,457 (8,850,977, 12,638,919)	10,642,127 (8,222,106, 13,383,779)	1181.61 (978.38, 1390.89)	501.26 (387.29, 627.98)	−2.75 (–2.92, –2.58)
Male	7,399,279 (5,450,273, 9,252,517)	7,740,359 (5,634,331, 10,365,104)	1634.85 (1218.61, 2045.07)	750.39 (550.90, 997.91)	−2.51 (–2.73, –2.30)
Female	3,374,178 (2,734,453, 4,160,646)	2,901,768 (2,251,657, 3,679,391)	743.14 (605.54, 913.28)	268.83 (208.91, 340.98)	−3.25 (–3.38, –3.11)
M/F	2.19	2.67	2.20	2.79	
**Liver cancer**					
Both	3,294,864 (2,763,029, 3,879,589)	4,890,023 (3,905,089, 6,124,599)	334.52 (281.08, 393.14)	239.91 (191.98, 299.37)	−0.96 (–1.36, –0.56)
Male	2,462,152 (2,000,446, 3,012,119)	3,702,093 (2,805,347, 4,985,654)	483.97 (392.36, 591.53)	368.19 (279.67, 490.95)	−0.76 (–1.24, –0.28)
Female	832,712 (666,725, 1,010,058)	1,187,930 (924,053, 1,513,173)	177.47 (142.84, 214.58)	111.91 (87.16, 141.96)	−1.42 (–1.76, –1.07)
M/F	2.96	3.12	2.73	3.29	
**Esophagus cancer**					
Both	5,852,132 (4,841,614, 6,818,927)	6,898,666 (5,471,181, 8,553,366)	653.31 (543.18, 758.88)	317.18 (252.46, 392.42)	−2.31 (–2.46, –2.16)
Male	4,329,941 (3,531,563, 5,230,398)	5,612,572 (4,255,105, 7,150,022)	962.82 (790.96, 1153.47)	534.18 (407.77, 678.22)	−1.90 (–2.06, –1.74)
Female	1,522,191 (779,916, 1,927,326)	1,286,094 (796,894, 1,686,072)	348.83 (180.81, 438.66)	114.58 (70.78, 150.41)	−3.51 (–3.77, –3.25)
M/F	2.84	4.36	2.76	4.66	
**Pancreatic cancer**					
Both	1,120,353 (941,076, 1,306,509)	2,930,317 (2,301,049, 3,575,079)	123.16 (103.69, 143.27)	137.23 (108.15, 166.74)	0.36 (0.18, 0.54)
Male	693,239(555,482, 84,5950)	1,854,033 (1,382,248, 2,393,909)	151.33 (122.42, 183.53)	179.36 (134.98, 229.10)	0.55 (0.37, 0.73)
Female	427,114(339,532, 530,296)	1,076,284 (806,636, 1,392,992)	95.37 (76.11, 118.14)	96.89 (72.71, 125.18)	0.07 (–0.12, 0.25)
M/F	1.62	1.72	1.59	1.85	
**Colorectal cancer**					
Both	3,565,196 (3,027,610, 4,106,701)	6,848,390 (5,513,407, 8,284,228)	390.63 (333.24, 448.92)	331.73 (267.78, 400.70)	−0.54 (–0.68, –0.41)
Male	2,039,861 (1,602,505, 2,481,120)	4,488,271 (3,427,062, 5,852,471)	454.10 (360.34, 547.99)	452.83 (349.19, 585.28)	−0.02 (–0.16, 0.12)
Female	1,525,334 (1,200,993, 1,884,418)	2,360,118 (1,798,152, 3,027,363)	334.19 (263.37, 410.15)	220.01 (167.51, 282.02)	−1.35 (–1.55, –1.15)
M/F	1.34	1.90	1.36	2.06	
**Gallbladder and biliary tract cancers**					
Both	452,349 (341,842, 581,398)	857,504 (601,927, 1,121,113)	52.61 (39.99, 67.15)	40.38 (28.21, 52.61)	−0.85 (–1.01, –0.69)
Male	221,390 (145,097, 311,194)	459,793 (269,773, 651,240)	52.86 (34.73, 75.75)	45.57 (26.80, 63.82)	−0.51 (–0.75, –0.28)
Female	230,959 (158,402, 322,844)	397,711 (248,642, 540,089)	52.85 (36.17, 73.76)	35.83 (22.37, 48.65)	−1.24 (–1.44, –1.04)
M/F	0.96	1.16	1.00	1.27	

Abbreviations: AAPC, average annual percentage change; ASDR, age‐standardized disability‐adjusted life year rates; CI, confidence interval; DALY, age‐standardized disability‐adjusted life year; UI, uncertainty interval.

### 3.2 Analysis of Gender Differences

In China, the burden of gastrointestinal tumors exhibits significant gender differences, with males experiencing a notably higher burden than females. Among the six gastrointestinal tumors, the gender disparities in esophageal, liver, and gastric cancers are particularly pronounced; the age‐standardized incidence rates (ASIR), age‐standardized mortality rates (ASMR), and age‐standardized disability‐adjusted life year rates (ASDR) gender ratios for esophageal cancer are 3.87, 4.12, and 4.66, respectively, in 2021. The ASIR, ASMR, and ASDR gender ratios for liver cancer are 2.93, 2.71, and 3.29, respectively, and for gastric cancer, they are 2.92, 2.71, and 2.79, respectively (Table 1). The time trend analysis revealed that the gender differences for most gastrointestinal tumors have shown an expanding trend from 1990 to 2021, particularly esophageal cancer, whose ASMR sex ratio increased from 2.50 in 1990 to 4.12 in 2021, a change of 64.8%.

### 3.3 Age‐Specific Analysis

**FIGURE 1:**
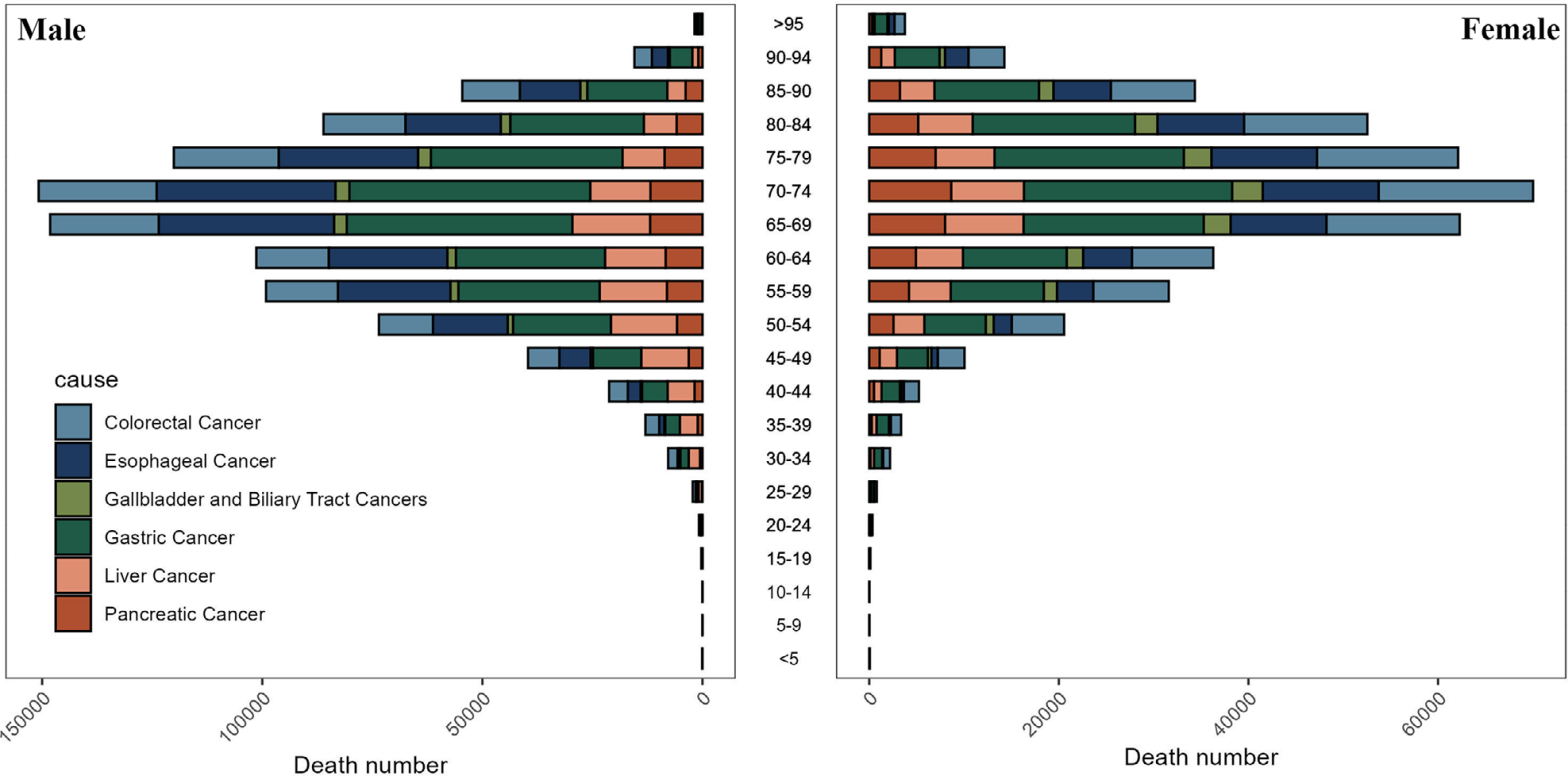
Distribution of absolute gastrointestinal tumor deaths in China by age group and sex in 2021.

**FIGURE 2:**
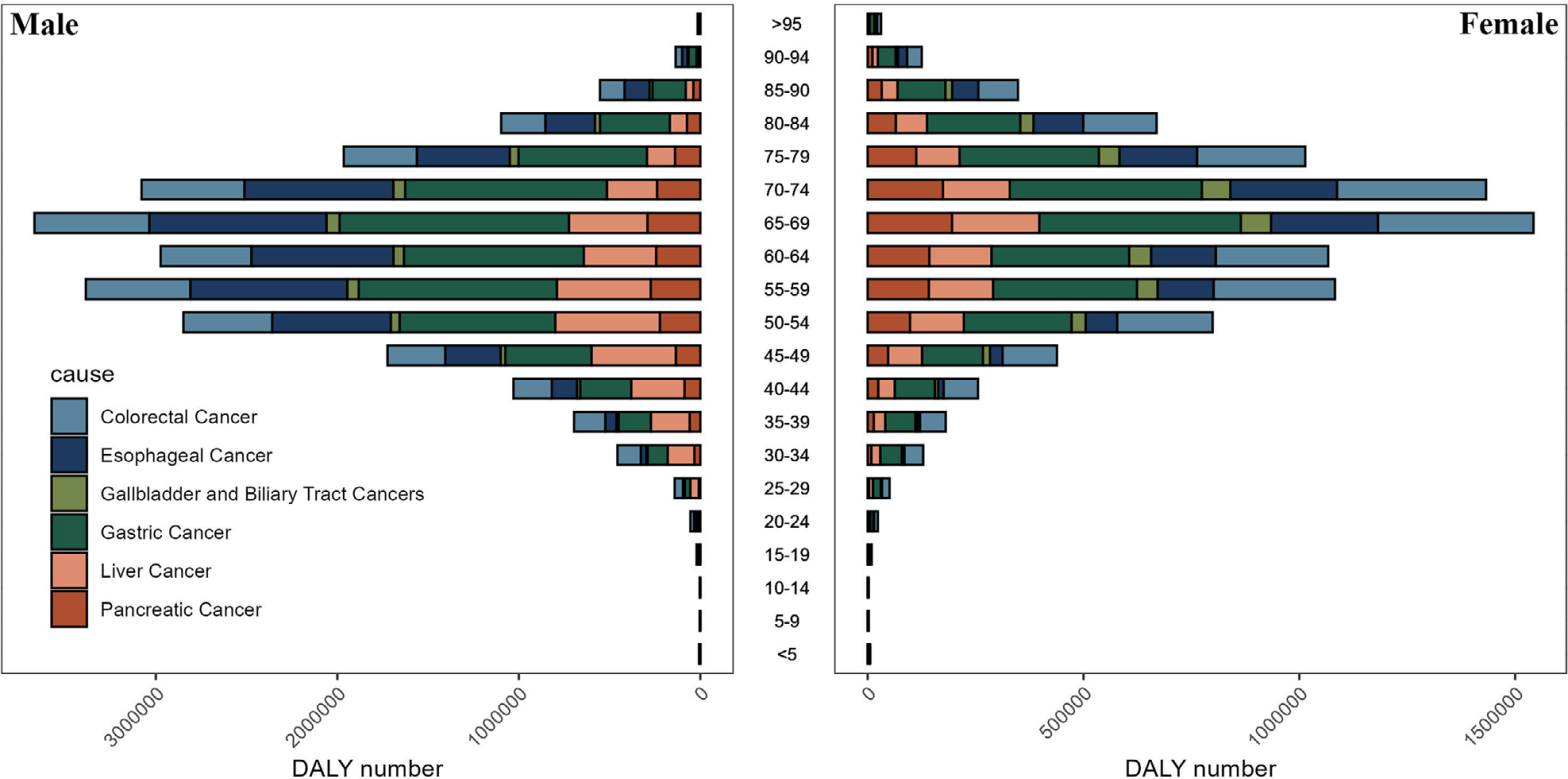
DALY distribution of gastrointestinal tumors in China by age group, sex in 2021.

**FIGURE 3:**
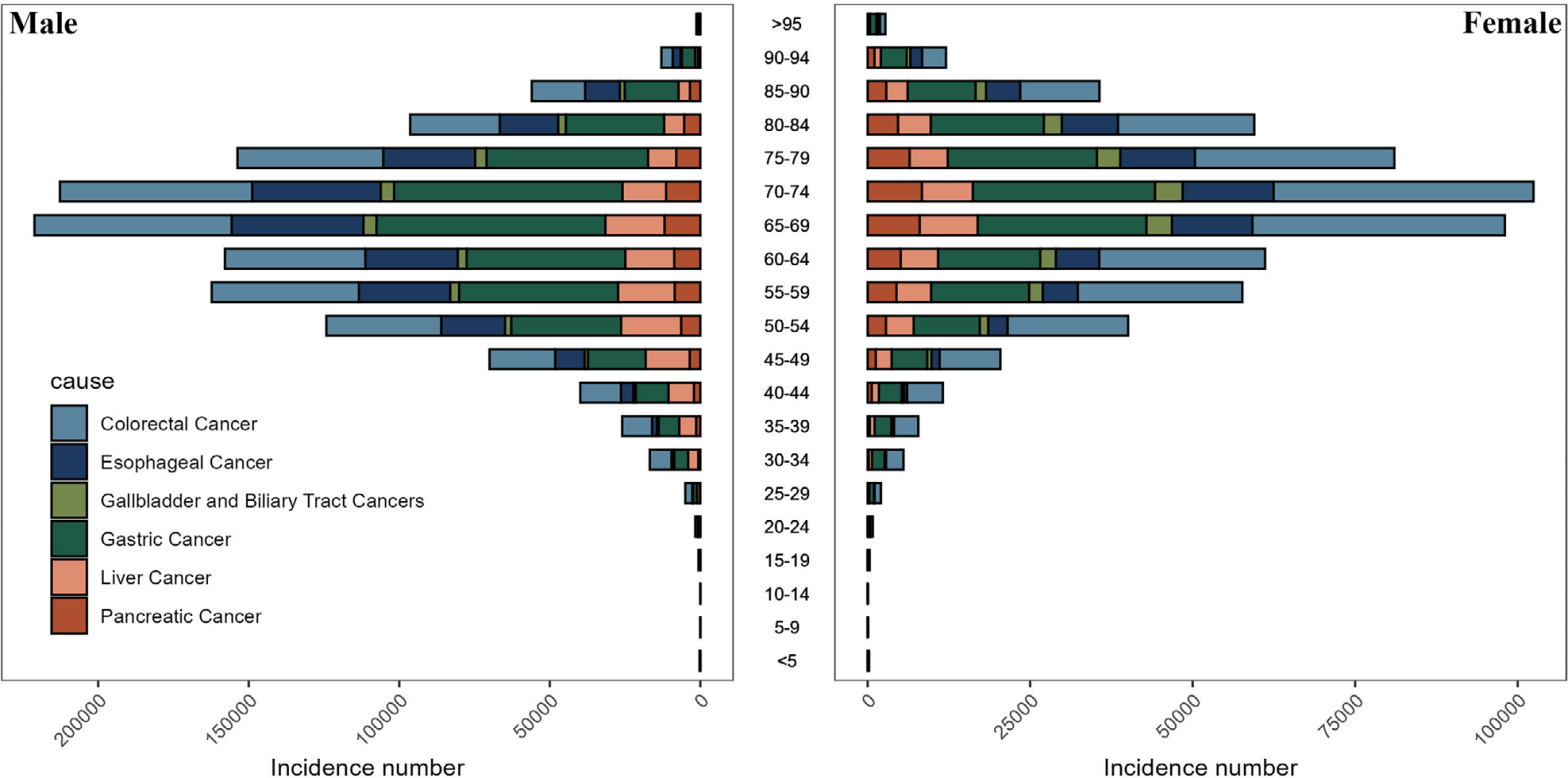
Incidence number distribution of gastrointestinal tumors in China by age group, sex in 2021.

Figure 1 illustrates the distribution of deaths across different genders and age groups from gastrointestinal tumors in China in 2021. It shows that, regardless of gender, the number of deaths is highest among individuals aged 70–74. For specific cancer types, the number of deaths from colorectal cancer peaks among those aged 75–79, while esophageal cancer, gallbladder and biliary tract cancer, pancreatic cancer, and gastric cancer peak among individuals aged 70–74. Liver cancer, on the other hand, peaks among those aged 65–69. Conversely, Figure 2 illustrates the distribution of disability‐adjusted life years lost for different gender age groups due to gastrointestinal tumors in China in 2021. In general, males and females aged 65–69 years lost the most disability‐adjusted life expectancy years, with gastric cancer accounting for the highest number of disability‐adjusted life years lost for both genders. As depicted in Figure 3, the highest age group for the incidence of gastrointestinal tumors in Chinese males in 2021 is 65–69 years, with gastric cancer being the most prevalent type. For females, the highest incidence of gastrointestinal tumors occurs in the 70–74 age group, where colorectal cancer is the most common.

### 3.4 Analysis of Temporal Trends in Cancer Burden

**FIGURE 4:**
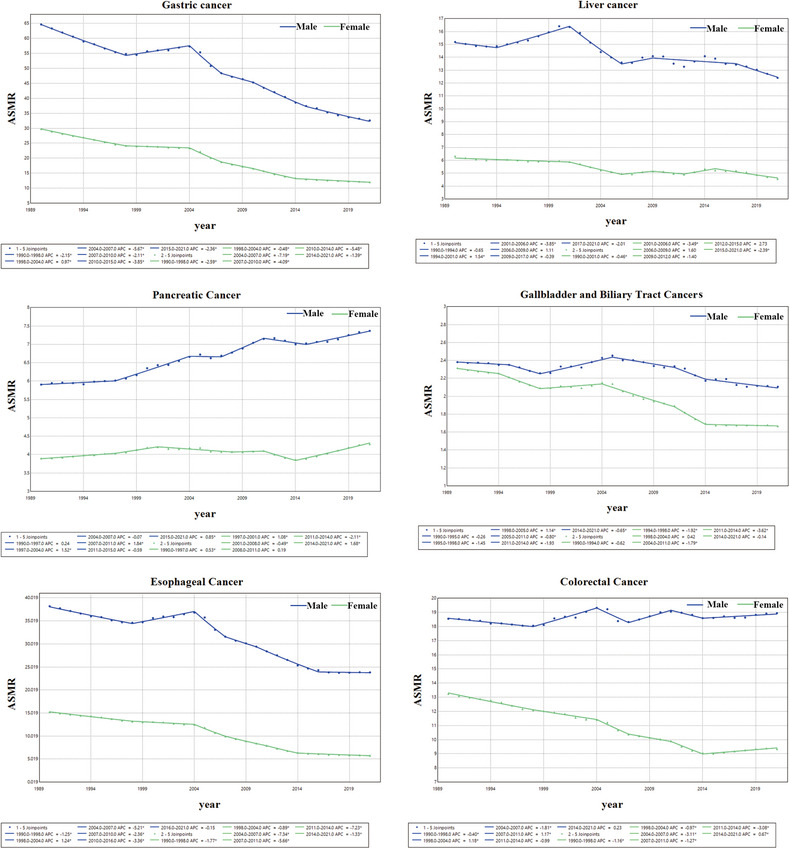
Trends in ASMR of gastrointestinal tumors in China, 1990–2021. The blue line represents males, while the green line represents females.

**FIGURE 5:**
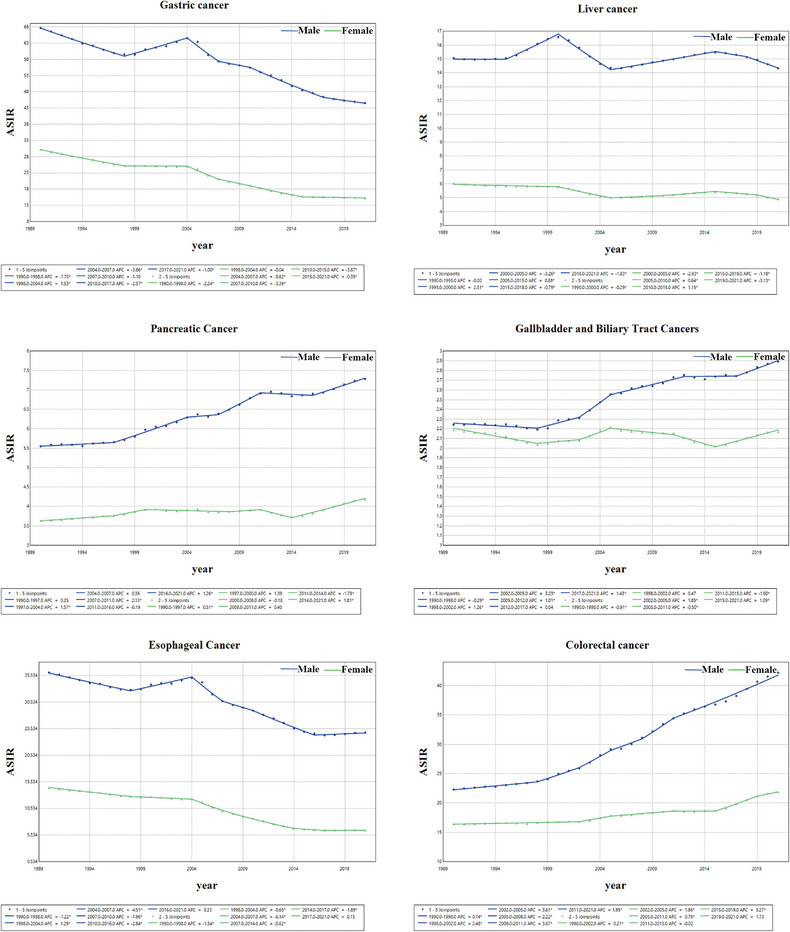
Trends in ASIR of gastrointestinal tumors in China, 1990–2021. The blue line represents males, while the green line represents females.

**FIGURE 6:**
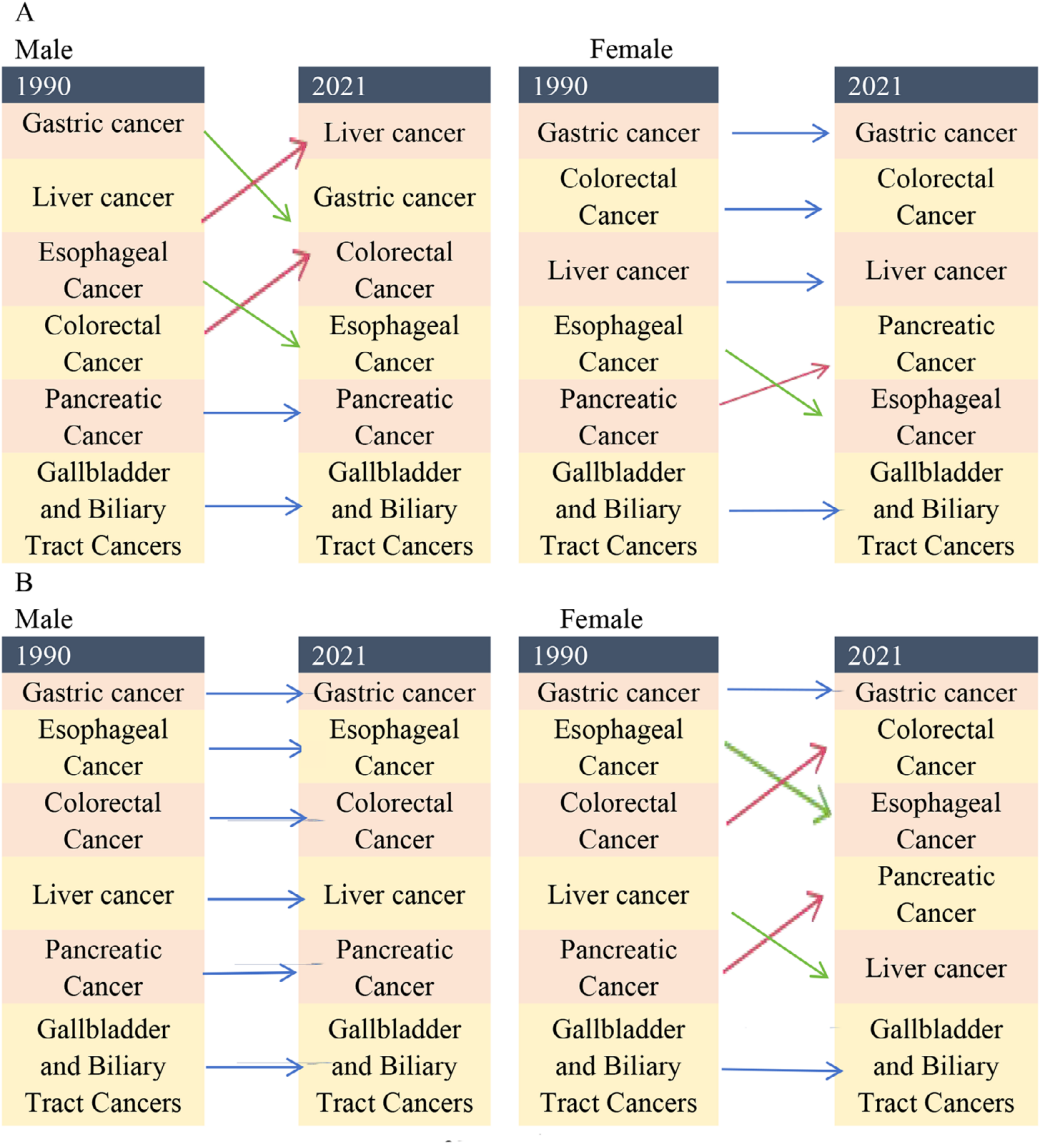
Ranking of gastrointestinal tumor deaths among males and females aged 15–49 (A) and ≥55 (B) in China in 1990 and 2021. The red line indicates an increase in rank. The green line indicates a decrease in rank. The blue line indicates no change in rank.

Between 1990 and 2021, the trends for ASIR, ASMR, and ASDR of gastric cancer displayed a decline, with an AAPC of –1.61 (95% CI, –1.74 to –1.49) (Table 1), –2.44 (95% CI, –2.62 to –2.26) (Table 2), and –2.75 (95% CI, –2.92 to –2.58) (Table 3). In particular, between 2004 and 2007, the decline in ASMR was most pronounced in males and females (Figure 4). Nevertheless, gastric cancer continued to be the leading gastrointestinal tumor in China in 2021, regarding ASMR and ASDR. In contrast, colorectal cancer had the most significant increase in ASIR, exhibiting an AAPC of 1.68 (95% CI, 1.55 to 1.80) (Table 1). Between 1990 and 1999, the ASIR of colorectal cancer in males exhibited slow growth, followed by a marked acceleration after 1999. In contrast, the ASIR in females remained relatively stable from 1990 to 2004, after which a gradual upward trend emerged (Figure 5), while its ASMR and ASDR showed declines of –1.11 (95% CI, –1.31 to –0.91) and –1.35 (95% CI, –1.55 to –1.15), respectively. Both liver and esophageal cancers displayed decreasing trends in ASIR, ASMR, and ASDR; however, liver cancer overtook gastric cancer as the gastrointestinal tumor with the highest mortality among Chinese males aged 15–49 years (Figure 6). Pancreatic cancer was unique among the six gastrointestinal tumors, as it showed rising trends in ASIR, ASMR, and ASDR, moving from fifth to fourth place in terms of deaths among Chinese females since 1990 (Figure 6). The ASIR of gallbladder and biliary tract cancers also increased, showing an AAPC of 0.40 (95% CI, 0.22 to 0.59) (Table 1), although both ASMR and ASDR exhibited declining trends. Throughout the analyzed period, gallbladder and biliary tract cancers have remained the gastrointestinal tumors with the fewest deaths in the Chinese population (Figure 6).

### 3.5 Sensitivity Analyses

Given the widely acknowledged limitations in the quality of Chinese cancer registry data in the early 1990s, we conducted sensitivity analyses to evaluate the robustness of our findings, with a specific focus on analyzing the AAPC in the ASIR, ASMR, and ASDR for six major gastrointestinal cancers in China from 2000 to 2021. The results demonstrated that the AAPC observed during the 2000–2021 period were generally consistent with the overall trends identified for the 1990–2021 period (Table S1).

## 4 Discussion

This study comprehensively analyzed the absolute and relative burden, along with temporal trends of gastrointestinal tumors in China from 1990 to 2021. In 2021, an estimated 1.96 million incident cases, 1.35 million deaths and 33.07 million DALYs were recorded for gastrointestinal tumors in China. Colorectal cancer had the highest incidence, while gastric cancer had the highest mortality burden. The ASIR increased for colorectal, pancreatic, gallbladder and biliary tract cancers, but decreased for other gastrointestinal tumors. With the exception of pancreatic cancer, ASMR and ASDR decreased for all five remaining gastrointestinal tumors.

Although the peak mortality for both males and females in 2021 occurred in the 70–74 age group, the incidence peaks for six gastrointestinal tumors varied by sex: males exhibited a peak at 65–69 years, while females showed a peak at 70–74 years. The delayed incidence peak in females may be attributed to the protective effects of estrogen, which could suppress oncogene expression, enhance mucosal protection, or modulate immune responses, thereby reducing tumor risk during reproductive years. However, post‐menopause, declining estrogen levels may contribute to an increased incidence, resulting in a later concentration of tumor onset in female [9]. Due to metabolic and genetic factors, males are more prone to metabolic dysregulation, which may accelerate oncogenesis. Additionally, males are more frequently exposed to modifiable risk factors such as smoking, excessive alcohol consumption, and occupational hazards, leading to a cumulative exposure effect that results in an earlier incidence peak for gastrointestinal tumors compared to females [10, 11, 12].

Colorectal cancer is the most common gastrointestinal tumor worldwide, including in China, as indicated by Globocan 2022 statistics [3, 13]. Preliminary research suggests a positive correlation between the Socio‐demographic Index (SDI), a metric of regional development, and colorectal cancer incidence. The incidence rate of colorectal cancer in high and very high SDI countries is approximately three times higher than in low and middle SDI countries [13]. The dietary and lifestyle changes brought about by China's rapid economic development are likely the primary contributing factors to the country's elevated incidence rate of colorectal cancer. Risk factors for colorectal cancer include a high‐fat diet [14], excessive red and processed meat consumption [15], sedentary lifestyle, smoking [16] and alcohol use. Given this, population‐based early colorectal cancer screening, dietary modification, improved surgical techniques, radiotherapy, chemotherapy, targeted therapy, and palliative care for colorectal cancer in China are important for improving the survival rate of colorectal cancer.

The downward trend observed in ASIR, ASMR, and ASDR for gastric cancer is likely to be a consequence of China's economic development. This development has led to improvements in the living standards of the population, as well as significant enhancements in living environments and public sanitation. Consequently, the infection rate of Helicobacter pylori (H. pylori), an established cause of gastric cancer, has been effectively reduced [17]. Nevertheless, in 2021, gastric cancer continued to be the most lethal type of gastrointestinal cancer in China. This is primarily attributable to the absence of distinct early symptoms and the rarity with which it is detected before clinical onset. Consequently, the mortality rate for this cancer remains high, with survival rates ranging from 20% to 40% in most countries [18, 19]. As a country with a high incidence of gastric cancer, the Republic of Korea (ROK) initiated a nationwide gastric cancer screening program in 1999. The results of this project indicated a 21% decrease in the overall mortality rate among the ROK population [20]. In comparison with Japan and the ROK, China's National Cancer Screening Program (CanSPUC), which was initiated in 2012, is comparatively late but is undergoing rapid improvement. CanSPUC employs a two‐step screening strategy for gastric cancer, initially using a questionnaire‐based risk assessment tool to identify high‐risk individuals, followed by endoscopic examination in designated hospitals, thereby contributing significantly to reducing the incidence and mortality rates of gastric cancer [21]. A multicenter cohort study in high‐risk areas for upper gastrointestinal cancer in China demonstrated that a single endoscopic screening reduced the mortality rate of non‐cardia gastric cancer by 61% in males and 64% in females [22]. In order to achieve optimal outcomes in the future, it is imperative to consider the implementation of additional screening procedures and the exploration of more cost‐effective screening strategies.

The incidence and mortality rates of liver cancer exhibit significant heterogeneity across different countries. A recent increase in these rates has been observed in numerous regions worldwide, including the United States, Australia, and most European countries. Conversely, a decrease in liver cancer incidence has been documented in certain Asian countries, such as the ROK, Japan, and China [13]. In China, the observed decrease in liver cancer incidence can be primarily attributed to the expansion of vaccination programs and the successful elimination or suppression of viral hepatitis [23, 24]. However, when compared to the analysis of the burden of gastrointestinal tumors in China based on GBD 2019 [25], it was observed that liver cancer jumped from the fourth to the third most common cancer type in terms of deaths among females aged 15–49 years in China in 2021. This observation underscores the necessity for enhanced screening and surveillance of liver cancer in high‐risk individuals in the future to improve the clinical outcomes of patients with liver cancer [26].

Of the six gastrointestinal tumors in China, pancreatic cancer is the only one for which both the ASIR and ASMR are increasing. This distinction warrants focused attention. Similar to gastric cancer, it does not manifest early symptoms that would allow for specific detection. The majority of patients diagnosed with pancreatic cancer have metastatic or locally advanced disease, which precludes the possibility of radical surgery. Moreover, the five‐year survival rate for patients with pancreatic cancer is approximately 10% [19]. Several key modifiable factors for pancreatic cancer have been identified, including smoking, obesity, diabetes, and alcohol consumption [27]. Smoking represents the most significant modifiable carcinogen. A meta‐analysis revealed that smokers exhibited a 1.74‐fold (95% CI, 1.61 to 1.87) increased risk of developing pancreatic cancer in comparison to non‐smokers [28]. Moreover, diabetes has been identified as an independent risk factor for pancreatic cancer in Asian populations, with studies demonstrating that diabetes increases the risk of developing pancreatic cancer by 1.8 times [29]. A multicenter case‐control study from China also indicates that diabetes is a significant risk factor contributing to the increasing burden of pancreatic cancer in China, with an odds ratio of 2.69 (95% CI, 1.51 to 4.77) [30]. Currently, clinical screening programs for the early detection of pancreatic cancer include the following: computed tomography (CT), magnetic resonance imaging (MRI), endoscopic ultrasonography (EUS), endoscopic retrograde cholangioscopy (ERCP), and magnetic resonance cholangioscopy (MRCP) [31, 32]. However, the sensitivity of these methods in screening patients with early‐stage pancreatic cancer remains relatively low. Consequently, there is a necessity for the implementation of more authoritative screening measures to provide support in the future.

China exhibits among the highest global incidence rates of esophageal cancer, anchoring the distinct “Central Asian Esophageal Cancer Belt” that extends from northern Iran through Central Asia to northwestern China [33]. Two main subtypes of esophageal cancer, adenocarcinoma (AC) and squamous cell carcinoma (SCC), account for the vast majority of cases, with SCC being the predominant form in China [34]. Elevated cancer risk has been observed to be associated with high‐temperature cooking, the curing of meat products, and the consumption of leftover foodstuffs [35]. Recent studies have demonstrated improved esophageal cancer survival rates in China over recent decades, primarily attributed to expanded endoscopic screening in high‐incidence regions [36, 37]. However, the invasive nature of endoscopy, its high cost, suboptimal patient compliance, and demanding sterilization requirements limit its feasibility for large‐scale screening of asymptomatic populations [38]. Hence, there is an imperative for further exploration of more sensitive and specific screening methods that are less harmful and more acceptable in the future.

Despite gallbladder and biliary tract cancers representing cancers with the lowest disease burden among gastrointestinal tumors in China, they remain a significant concern given their increasing incidence. Gallbladder and biliary tract cancers, the most prevalent biliary tract malignancy, are difficult to detect early and carry a poor prognosis when diagnosed at advanced stages [39]. The management of risk factors constitutes the foundation of prevention and control strategies for gallbladder and biliary tract cancers. Research has indicated that a persistent infection of the gallbladder and biliary system, gallstones, has the potential to elevate the likelihood of developing gallbladder cancer [40]. Surgical resection is currently recognized as the only treatment with the potential to achieve the goal of eradicating gallbladder and biliary tract cancers. However, very few cases are amenable to resection, and most adjuvant treatments have very low response rates [41]. Consequently, there is an ongoing need for further discovery and validation of reliable biomarkers for screening, treatment selection, and prognosis.

This study has several strengths. Firstly, the most recent database from GBD 2021 was utilized, as it provides more reliable estimates of disease burden through the implementation of disease modelling by DisMod‐MR. Secondly, the study evaluated the prevalence of gastrointestinal tumors in China by comparing the demographic characteristics of patients across various age groups, sexes, and years. This analysis will inform the development of more rational public gastrointestinal cancer control measures in the future.

However, there are some limitations to our study. Firstly, we were unable to obtain data on different subtypes of gastrointestinal tumors, limiting further data analysis. Specifically, the GBD failed to differentiate intrahepatic cholangiocarcinoma (ICC) from liver cancer, which may bias liver‐cancer trend interpretation. Secondly, the dearth of disease burden estimation at the provincial level constitutes a significant impediment to the translation of research findings into targeted policies. Despite China's overall development, the presence of considerable economic disparities and divergent disease patterns among provinces engenders substantial limitations on the implementation of targeted policies.

## 5 Conclusion

In summary, the present study concentrated on the burden of gastrointestinal tumors and trends in China from 1990 to 2021, based on the GBD 2021. A comprehensive analysis reveals a substantial decline in the prevalence of gastrointestinal tumors in China. This phenomenon can be largely attributed to the nation's growing commitment to public health initiatives and the strategic implementation of vaccines. However, gender and age disparities in gastrointestinal tumor burden still persist, suggesting potential genetic, hormonal, or immunological underpinnings. These disparities may also be attributable to varying exposure levels to environmental risk factors between males and females. The implementation of tertiary prevention strategies for gastrointestinal tumors in key populations still needs to be intensified in the future to reduce the burden of incidence and mortality in male and older populations.

## Conflicts of Interest

The authors declare no conflicts of interest.

## Supporting information




**Supporting Table 1**: The AAPC of age‐standardized incidence, mortality, and disability‐adjusted life year rates from 2000 to 2021.
