# Supplementary material for: Disease Burden of Gastrointestinal Tumors in China From 1990 to 2021, an Analysis for the Global Burden of Disease Study 2021
Source: J Evid Based Med. 2025 Sep 20;18(3):e70072. doi: 10.1111/jebm.70072 (PMC12506945; doi:10.1111/jebm.70072)
Supplement: Supplementary file 1 — Supporting Table 1: The AAPC of age‐standardized incidence, mortality, and disability‐adjusted life year rates from 2000 to 2021. [file JEBM-18-0-s001.docx]

| Supplementary Table 1 The AAPC of age-standardized incidence, mortality, and disability-adjusted life year rates from 2000 to 2021 | | | |  |  |  |  |
| --- | --- | --- | --- | --- | --- | --- | --- |
| Cancer type | ASIR(95%CI) | ASMR(95%CI) | ASDR(95%CI) |  |  |  |  |
| **Gastric cancer** | -1.76(-1.90,-1.62) | -2.78(-3.04-2.53) | -3.06(-3.29,-2.84) |  |  |  |  |
| **Liver cancer** | -0.79(-0.94,-0.64) | -1.42(-2.22,-0.60) | -1.72(-2.59,-0.85) |  |  |  |  |
| **Esophagus cancer** | -1.92(-2.14,-1.70) | -2.45(-2.73,-2.17) | -2.75(-2.94,-2.56) |  |  |  |  |
| **Pancreatic cancer** | 0.70(0.38,1.02) | 0.46(0.08,0.84) | 0.37(0.13,0.61) |  |  |  |  |
| **colorectal cancer** | 2.11(1.86,2.37) | -0.36(-0.55 -0.18) | -0.39(-0.47 -0.25) |  |  |  |  |
| **Gallbladder and Biliary Tract Cancers** | 0.72(0.50,0.94) | -0.76(-1.25,-0.26) | -0.79(-0.99,-0.59) |  |  |  |  |
| Abbreviations: AAPC, average annual percentage change; ASIR, age-standardized incidence rates;ASMR, age-standardized mortality rates;ASDR,age-standardized disability-adjusted life year rates；CI,confidence interval. | | | | | | | |
